# Detection of inter-patient left and right bundle branch block heartbeats in ECG using ensemble classifiers

**DOI:** 10.1186/1475-925X-13-72

**Published:** 2014-06-05

**Authors:** Huifang Huang, Jie Liu, Qiang Zhu, Ruiping Wang, Guangshu Hu

**Affiliations:** 1 Department of Biomedical Engineering, School of Computer and Information Technology, Beijing Jiaotong University, 3 Shang Yuan Cun, Hai Dian District, Beijing, China; 2 Department of Biomedical Engineering, School of Medicine, Tsinghua University, Beijing, China

**Keywords:** Heartbeat classification, Left bundle branch block (LBBB), Right bundle branch block (RBBB), Independent component analysis (ICA), Linear discriminant classifier, Support vector machine (SVM), Ensemble

## Abstract

**Background:**

Left bundle branch block (LBBB) and right bundle branch block (RBBB) not only mask electrocardiogram (ECG) changes that reflect diseases but also indicate important underlying pathology. The timely detection of LBBB and RBBB is critical in the treatment of cardiac diseases. Inter-patient heartbeat classification is based on independent training and testing sets to construct and evaluate a heartbeat classification system. Therefore, a heartbeat classification system with a high performance evaluation possesses a strong predictive capability for unknown data. The aim of this study was to propose a method for inter-patient classification of heartbeats to accurately detect LBBB and RBBB from the normal beat (NORM).

**Methods:**

This study proposed a heartbeat classification method through a combination of three different types of classifiers: a minimum distance classifier constructed between NORM and LBBB; a weighted linear discriminant classifier between NORM and RBBB based on Bayesian decision making using posterior probabilities; and a linear support vector machine (SVM) between LBBB and RBBB. Each classifier was used with matching features to obtain better classification performance. The final types of the test heartbeats were determined using a majority voting strategy through the combination of class labels from the three classifiers. The optimal parameters for the classifiers were selected using cross-validation on the training set. The effects of different lead configurations on the classification results were assessed, and the performance of these three classifiers was compared for the detection of each pair of heartbeat types.

**Results:**

The study results showed that a two-lead configuration exhibited better classification results compared with a single-lead configuration. The construction of a classifier with good performance between each pair of heartbeat types significantly improved the heartbeat classification performance. The results showed a sensitivity of 91.4% and a positive predictive value of 37.3% for LBBB and a sensitivity of 92.8% and a positive predictive value of 88.8% for RBBB.

**Conclusions:**

A multi-classifier ensemble method was proposed based on inter-patient data and demonstrated a satisfactory classification performance. This approach has the potential for application in clinical practice to distinguish LBBB and RBBB from NORM of unknown patients.

## Background

**Figure 1:**
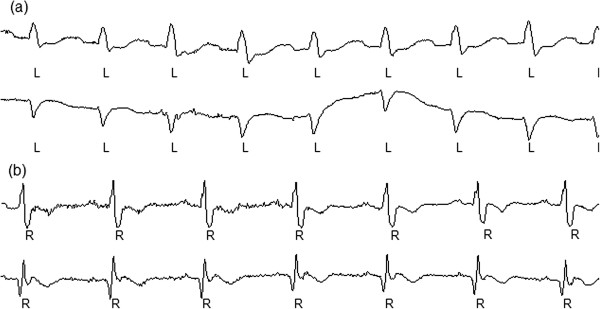
**Typical ECGs of LBBB and RBBB from two leads. (a)** The ECG of LBBB from two leads. Upper: lead A; lower: lead B. **(b)** The ECG of RBBB from two leads. Upper: lead A; lower: lead B.

Under normal circumstances, excitation from the sinoatrial node controls the heart rhythm. An abnormality in the sinus rhythm leads to arrhythmia, which refers to abnormalities in the rate, rhythm, site of origin, and conduction of the cardiac electrical pulse. When disorders occur in specific intraventricular conduction fibers, the repolarization wave must travel through the slower muscle-muscle conduction to reach the ventricles. Classic disorders related to conditions that involve different conduction bundle branches include left bundle branch block (LBBB) and right bundle branch block (RBBB). Electrocardiogram (ECG) can be used to measure and record cardiac electrical activities and thus can provide important information on cardiac functions. ECG has been used as a standard diagnostic tool to analyze arrhythmia. Typical ECGs of LBBB and RBBB are shown in Figure 1. LBBB and RBBB usually have a minimal impact on blood pumping. However, these conditions can change the ECG and mask the ECG changes that reflect disease conditions, such as ischemia. In some cases, these conduction abnormalities indicate important underlying pathological conditions, such as a new occurrence of LBBB resulting from acute anterior ischemia or a new occurrence of RBBB caused by pulmonary embolism [1]. Some studies have reported that LBBB and RBBB have important prognostic value. The prevalence of LBBB increases the risk of coronary heart disease and ventricular myocardial infarction. The prevalence of RBBB is associated with cardiovascular diseases, such as arterial hypertension, heart failure, coronary disease, and pulmonary embolism. Individuals with comorbid cardiovascular diseases and LBBB or RBBB have a higher risk of mortality compared with individuals without LBBB or RBBB. Consequently, the timely detection of LBBB and RBBB is critical in clinical treatment [2-4].

LBBB and RBBB manifest as a series of heartbeats with abnormal intervals and morphologies. Heartbeat classification is an important step in identifying LBBB and RBBB because they can be determined through the classification of continuous heartbeats [5]. For accurate qualitative and quantitative analyses of arrhythmias, a Holter monitor can be used to record thousands of heartbeats. As a result, heartbeat classification is a relatively time-consuming process. With the aid of a computer, automatic heartbeat classification can be achieved, and new algorithms can be developed to improve the classification performance.

In clinical practice, a heartbeat classification system is typically constructed using labeled ECG data. It is then used to predict the types of heartbeats in the ECG recordings of unknown clinical patients. The predictive performance of a heartbeat classification system is closely related to the dataset division method used in system construction. To date, a heartbeat-oriented dataset has been adopted in most studies [6-15], without consideration of the subject to whom the heartbeat belongs (i.e., recording number). Consequently, data in the training and testing sets may originate from the same patient, leading to optimistic classification results [16]. The heartbeat classification systems built on such datasets have poor predictive capability. Some training sets include both global and local training sets. A global training set is obtained from many patients’ ECG recordings but is independent of the testing set. Local training sets use a small section from the beginning of each patient’s recording as the training samples for the subsequent test of this recording to improve the classification performance. This technique is called patient-adaptive heartbeat classification [17-19]. Because the local training and testing sets originate from the same patient, this method presents a very high accuracy rate in heartbeat classification. However, it requires denotation by experts and cannot be fully automated. The two classification methods discussed are for intra-patient classification. To improve the predictive capability of heartbeat classification systems and achieve automatic classification of heartbeats, a system should be constructed on the basis of independent training and testing sets (the training set and testing set originate from different individuals), namely, inter-patient classification. In this case, heartbeat classification is more challenging.

The Association for the Advancement of Medical Instrumentation (AAMI) has proposed standards for the performance evaluation of arrhythmia analysis algorithms [20]. The MIT-BIH Arrhythmia Database [21] is the most commonly used database for testing algorithms. The AAMI has classified the 15 types of heartbeats identified in the MIT-BIH Arrhythmia Database into five classes. The classes include heartbeats that originate in the sinoatrial node (N), supraventricular ectopic beat (S), ventricular ectopic beat (V), fusion heartbeat (F), and unknown beat type (Q). Recently, progress has been made in studies on inter-patient heartbeat classification methods based on the AAMI standards [5,16,22-25].

In addition to the normal beat (NORM), class N beats primarily include LBBB and RBBB. Based on the AAMI standards, the current inter-patient heartbeat classification usually only distinguishes class N beats from other classes of heartbeats, but it cannot further distinguish LBBB and RBBB from NORM. A large number of the previous studies that involved LBBB and RBBB classification only investigated intra-patient classification [7-13]. Despite good results, it is difficult to utilize these methods to predict LBBB and RBBB in unknown patients’ ECG recordings. Yeh et al. [26-28] proposed several classification methods for LBBB and RBBB detection based on parametric features. However, their study did not explicitly use an independent training set, and it is not clear whether the methods belong to inter-patient classification. A few studies detected LBBB and RBBB based on inter-patient classification. Jekova et al. [8] employed parameter features and the kth nearest neighbor classifier for LBBB and RBBB detection. Mishra et al. [11] proposed a local fractal dimension based nearest neighbor classifier to detect LBBB and RBBB. However, the sensitivities of LBBB and RBBB in two studies were lower than 90%. Dokur et al. [29] represented an intersecting sphere (InS) network for classification using discrete wavelet transform. Despite a high performance, the number of NORMs was so small that it lowered the difficulty of classification. Therefore, the aim of this study was to propose an inter-patient heartbeat classification method to accurately distinguish LBBB, RBBB, and NORM in class N beats based on large datasets. Through our experimental observation, large variations were observed in heartbeats of the same type because of inter-individual variations; meanwhile, the morphologies and RR intervals of LBBB and RBBB were similar to those of NORM. LBBB and NORM are particularly difficult to distinguish. As a result, it is difficult to obtain a satisfactory classification of these three types of heartbeats based on independent training and testing sets.

**Figure 2:**
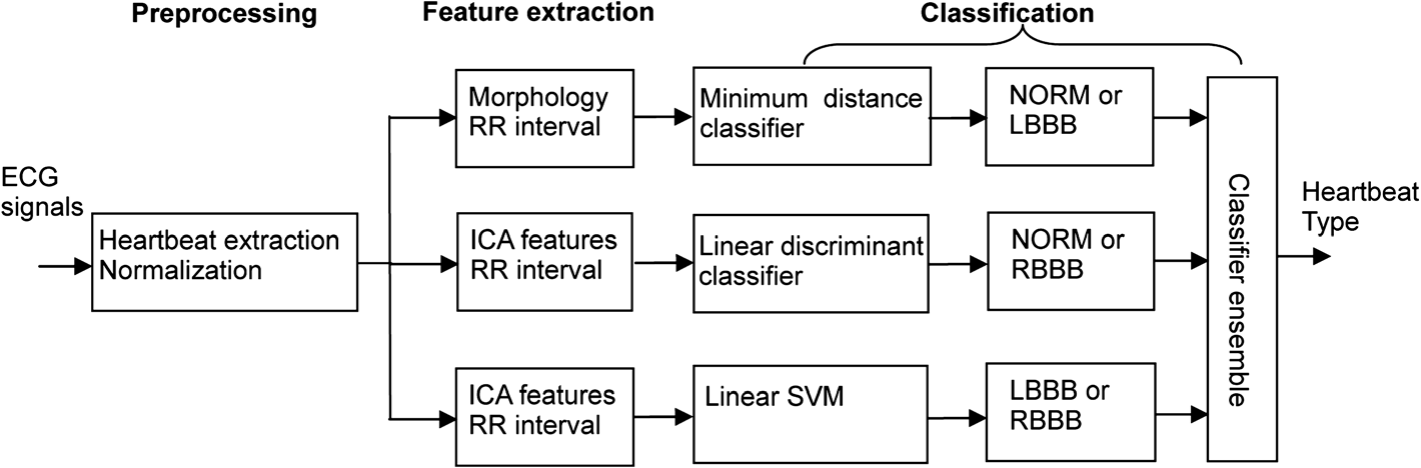
**An overview of the classification of LBBB, RBBB, and NORM using ensemble classifiers.** The heartbeats were extracted from ECG signals and normalized in the preprocessing stage. Then, they were processed in parallel through the three branches, each of which included feature extraction and classification. Each classifier provided two possible class labels for the tested heartbeats, and the final type of the heartbeat was determined using a majority voting strategy through the combination of the class labels from the three classifiers.

The combination of multiple classifiers facilitates the integration of knowledge obtained from different classifiers to improve the accuracy of the ultimate classification [30,31]. To obtain improved integration of the classification results, the component classifiers must typically possess a good classification performance with simultaneously significant differences [32]. This study proposed a heartbeat classification method through the integration of three different classifiers. A high performance classification was achieved via the construction of an effective component classifier between each pair of heartbeat types (shown in Figure 2). A minimum distance classifier was constructed between NORM and LBBB. A weighted linear discriminant classifier was constructed between NORM and RBBB based on Bayesian decision making using posterior probabilities. A linear support vector machine (SVM) was constructed between LBBB and RBBB. Each classifier provided two possible class labels for the tested heartbeats, and the final classification of the examined heartbeats was determined using a majority voting strategy through the combination of the class labels from the three classifiers. Each classifier was used with matching features to obtain better classification performance. The optimal parameters for the classifiers were selected using cross-validation of the training set. The effects of different lead configurations on the classification results were assessed, and the classification performance of these three classifiers was compared for the detection of each pair of heartbeat types. The study results indicated better classification performance with two-lead configurations compared with single-lead configurations. The proposed multi-classifier ensemble method showed a satisfactory classification performance.

## Methods

### ECG data

The datasets utilized in this study were obtained from the MIT-BIH Arrhythmia Database [21]. The data included forty-eight 30 minute ECG recordings collected from two leads (labeled here as leads A and B). There were 109,492 heartbeats, which were divided into 15 heartbeat types. Class N beats defined by the AAMI primarily include three heartbeat types, NORM, LBBB, and RBBB; thus, only three types of heartbeats were studied. In this study, recordings in the MIT-BIH Arrhythmia Database were divided as described in [5]. Consistent with the standard recommendation by the AAMI, four heartbeat recordings in which a paced beat was involved were discarded [5]. The remaining 44 recordings were divided into two datasets, DS1 and DS2, each of which contained 22 recordings. DS1 was utilized as the training set, and DS2 was utilized as the testing set. The numbers of the three heartbeat types in the two datasets are shown in Table 1.

**Table 1:** Distribution of heartbeat types in the two independent datasets

**Dataset**	**NORM**	**LBBB**	**RBBB**	**Total**
DS1	38104	3949	3783	45836
DS2	36444	4125	3476	44045
Total	74548	8074	7259	89881

Dataset DS1 includes data from the following recordings: 101, 106, 108, 109, 112, 114, 115, 116, 118, 119, 122, 124, 201, 203, 205, 207, 208, 209, 215, 220, 223, and 220. Dataset DS2 includes data from the following recordings: 100, 103, 105, 111, 105, 7, 11, 121, 123, 200, 202, 210, 212, 213, 214, 219, 221, 222, 228, 231, 233, and 234. Four recordings (102, 104, 107, and 217) that contained pace beats were not included in DS1 or DS2.

### Overview of the proposed method

The heartbeat classification process is usually divided into three parts: preprocessing, feature extraction, and classification. Figure 2 shows the stages of the proposed method for heartbeat classification in this study.

In the preprocessing stage, the heartbeats were extracted from ECG signals using the R peak position provided by the MIT-BIH Arrhythmia Database and then normalized.

The preprocessed heartbeats were then processed in parallel through the three branches, each of which included feature extraction and classification. The classification performance depends largely on the design of the classifier. Because of the different extents of overlap and different distributions of each pair of heartbeat types, three classifiers were used in this study: the minimum distance classifier, the linear discriminant classifier, and the linear SVM. Feature extraction also has a great impact on the performance of a classifier. A classifier can only achieve better performance when appropriate features are applied. The input features of the minimum distance classifier included the morphology of the preprocessed heartbeat and the RR interval. The input features of the linear discriminant classifier and the linear SVM included the independent component analysis (ICA) features and the RR interval. Each classifier was built based on each pair of heartbeat types in the training set DS1 (the minimum distance classifier between NORM and LBBB; the linear discriminant classifier between NORM and RBBB; and the linear SVM between LBBB and RBBB).

Each classifier subsequently produced two possible type labels for a test heartbeat because each classifier was trained between each pair of heartbeat types in the training set. In other words, each classifier was concerned with the classification of two types of heartbeats. Another type of heartbeat not involved in the training procedure was assigned to the type label by the classifier.

Finally, the preprocessed test heartbeats, including NORM, LBBB, and RBBB, in the dataset DS2 were assigned to the type labels using a majority voting strategy through the combination of the type labels from the three classifiers. Multi-class classification was implemented through a one-against-one classification method, which used three distinct classifiers with matching features. The preprocessing, construction of the three classifiers, and classification of the test heartbeats are described in additional detail below.

### Preprocessing

Extraction of the heartbeats first requires QRS wave detection. Currently, many methods are available for QRS detection with accuracy rates greater than 99.5% [33-35]. The focus of this study was on heartbeat classification, not on QRS detection. Therefore, the reference points provided by the annotated files in the MIT-BIH Arrhythmia Database [21] were used directly. The ECG sampling frequency was 360 Hz. Each heartbeat sample was segmented from 0.278 s before to 0.278 s after the R wave and included 200 points. Thus, the sample included a complete heartbeat signal, which was composed of the QRS complex, the P wave, and the T wave. To reduce incorrect decisions as a result of signal amplitude deviation generated by the apparatus and individual variation, each heartbeat sample was subtracted by the mean and then divided by the standard deviation. Thus, a series of zero-mean, unit-standard-deviation normalized waveforms were produced, which represented preprocessed heartbeats. Prior to heartbeat extraction and normalization, the ECG signal was not denoised by a filtering method because filter denoising could cause the distortion of heartbeat morphology; thus, the information useful for classification in the heartbeats would be lost and subsequent heartbeat classification could be affected.

### Construction of the minimum distance classifier

**Figure 3:**
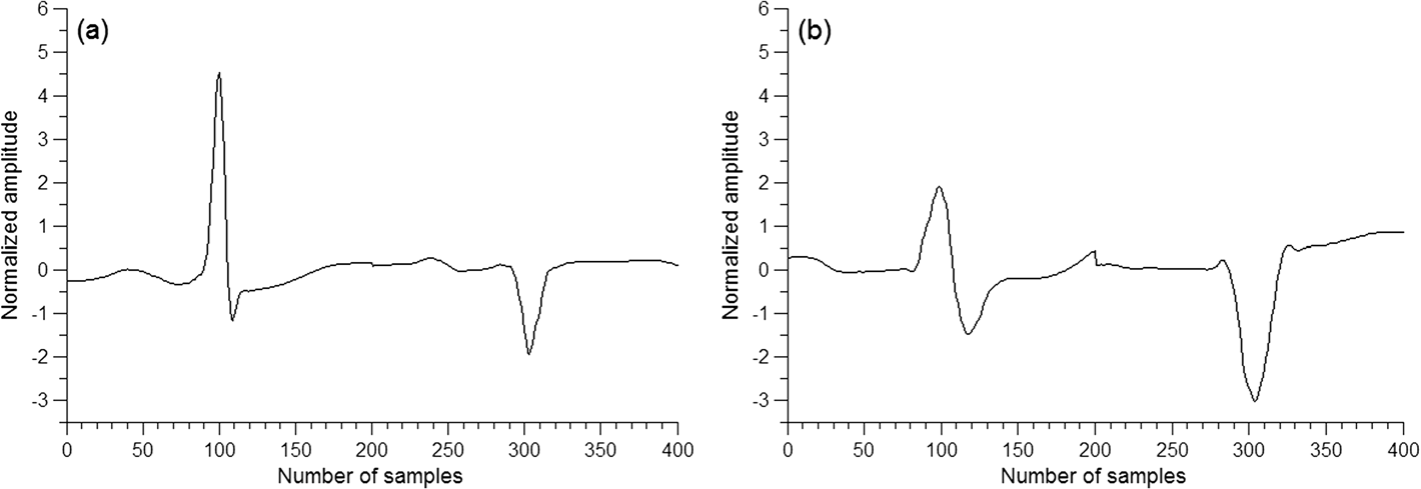
**The two-lead heartbeat mean vectors of NORM and LBBB in the training set. (a)** The mean vector of NORM. **(b)** The mean vector of LBBB.

A minimum distance classifier was constructed between NORM and LBBB in the training set DS1. The feature vector used here consisted of the preprocessed heartbeats of the two leads and the RR interval, i.e., a 401-dimensional vector.Because the majority of heartbeats are NORM in various forms, and some NORMs are close to LBBB morphology, NORM and LBBB overlap heavily with many heartbeats at the border between the two types. To exclude the impact of these border heartbeats, a minimum distance classifier was used for the classification of NORM and LBBB with the consideration of the centroids (mean vectors) of two heartbeat types. Considering NORM and LBBB as the two clusters, the mean vectors, or the centroids, of NORM and LBBB heartbeats in the training set were first calculated. The 400-dimension heartbeat mean vectors of NORM and LBBB are shown in Figure 3 (not including the RR interval).

The distances from any heartbeat of the testing set to the two centroids were then calculated and compared, and the heartbeat was classified in the type with the closer centroid. The minimum distance classifier is relatively simple and does not require parameter selection.

### Construction of the weighted linear discriminant classifier

A weighted linear discriminant classifier was constructed between NORM and RBBB in the training set DS1 based on Bayesian decision making using posterior probabilities. In order to achieve an improved classification performance with a reduced number of feature dimensions, the feature vector used was composed of the ICA-based features of the two leads and the RR interval, i.e., a 201-dimensional vector.

#### Application of ICA for extraction of heartbeat features

ICA is a statistical method that can separate a set of random variables into statistically independent potential components. It estimates the independent components from the data itself, extracts the essential features, and simultaneously reduces the dimensions [36]. Compared with principal component analysis (PCA), ICA captures not only second order statistics but higher order statistics and contains more feature information. Therefore, ICA has become a highly useful feature extraction method. The application of ICA to heartbeat classification has produced satisfactory results [15,37,38]. In this study, the use of ICA for the extraction of heartbeat features could prevent feature vectors from diverging in the feature space and facilitate the classification performance of the linear discriminant classifier.

**Figure 4:**
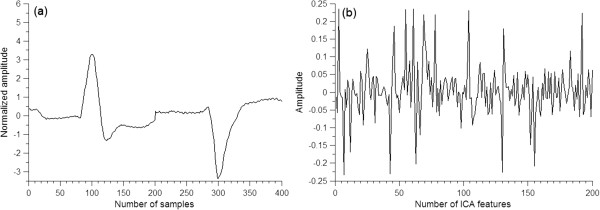
**A preprocessed two-lead heartbeat and its ICA features. (a)** A preprocessed LBBB heartbeat. **(b)** The ICA features of the heartbeat shown in (a).

Many algorithms have been proposed for implementing ICA. Hyvärinen’s FastICA algorithm [39], which uses a fixed point iteration scheme, provides a great advantage in the rapid estimation of ICA because it is much faster compared with the traditional gradient descent methods for ICA. Therefore, the FastICA algorithm was used to calculate the independent components in this study. Because ICA for heartbeat feature extraction was also used in the subsequent classifier construction between LBBB and RBBB, 180 NORMs, 200 LBBB beats, and 240 RBBB beats were extracted separately from the training set of 22 records. Ten NORMs were extracted from each of the 18 records, 100 LBBB beats from each of the 2 records, and 80 RBBB beats from each of the 3 records. This extraction resulted in 620 heartbeats from which 100 independent components were calculated in order using the FastICA algorithm. In the final step, the heartbeat samples were projected onto the independent components to produce 100 ICA-based features (i.e., each heartbeat sample was multiplied by the pseudo-inverse of the matrix that consisted of 100 independent components and obtained 100 ICA-based features). The feature vector used here was composed of 200 ICA features of the two leads and one RR interval. Figure 4(a) shows a preprocessed two-lead heartbeat, and Figure 4(b) shows the ICA features of the heartbeat in Figure 4(a).

#### Construction of the weighted linear discriminant classifier

Because the quantity of NORM is far greater compared with the quantity of RBBB in the heartbeat datasets, to prevent skewed classification results toward the majority class, a weighted linear discriminant classifier based on Bayesian posterior probability decision making, previously described in [5], was used to classify NORM and RBBB. This classifier assumes Gaussian distributions of two types of heartbeats with the same priori probability and assigns the test sample to the type with the maximum posterior probability. The classification performance is improved by eliminating the effects of the heartbeats with similar morphology at the border between the two types of heartbeats.

The linear discriminant classifier must first calculate the mean vector and covariance from the training set, and the covariance matrix ∑ is defined as [5]

(1) $$\Sigma =\sum_{i=1}^{C}w_{i}\sum_{k=1}^{N_{i}}\left(x_{\mathrm{ik}}-m_{i}\right){\left(x_{\mathrm{ik}}-m_{i}\right)}^{T}$$

where **x**_*ik*_ is the feature vector of heartbeat *k* in class *i*; *N*_*i*_ is the number of heartbeats in class *i*; **m**_*i*_ is the mean vector of class *i*; and *C* is the number of classes. The values of the weighting coefficients *w*_*i*_ of the two heartbeat types were experimentally determined in the training set.

After computing **m**_*i*_ and ∑ from the training set, assuming Gaussian distributions of the two types of training feature vectors and based on Bayesian decision making theory, the posterior probability of an unknown heartbeat feature vector **x** belonging to class *i* was calculated by

(2) $$P\left(i|x\right)=\frac{\exp\left[g_{i}\left(x\right)\right]}{\sum_{n=1}^{2}\exp\left[g_{n}\left(x\right)\right]}\;i=1,2$$

where

(3) $$g_{i}\left(x\right)=m_{i}^{T}\Sigma^{-1}x-\frac{1}{2}m_{i}^{T}\Sigma^{-1}m_{i}+\log\left(P\left(\omega_{i}\right)\right),\;i=1,2$$

*P*(*ω*_*i*_) is the priori probability, which is 0.5 for both NORM and RBBB. The unknown heartbeat can then be assigned to the type with the maximum posteriori probability estimated from Equation (2).

### Construction of the linear SVM

A linear SVM was constructed between LBBB and RBBB in the training set DS1. ICA was also used to extract heartbeat features, and the input feature vector consisted of the ICA features from two leads and the RR interval.

LBBB and RBBB are relatively easy to distinguish. Although the use of the previously described weighted linear discriminant classifier can yield a relatively good classification performance, the use of a linear SVM can yield even better classification performance.

SVM is a widely used classification method based on minimizing structural risk, with good generalization performance. SVM maps data in an original low-dimensional space to a high-dimensional feature space using a kernel function. The best separation surface in the high-dimensional feature space is determined to maximize the margin between the training data and the decision making border. The closest training data to the decision border constitute the support vectors [40,41].

The linear SVM performs substantially faster compared with the SVM with radial basis function (RBF) kernels and must only determine the penalty parameter; thus, the use of the linear SVM can improve the efficiency of training and classification. The classification performance of linear SVM depends largely on the choice of the penalty parameter *C*. The penalty parameter was experimentally determined to achieve the best classification performance, and liblinear-1.93 in the MATLAB environment was used for the SVM experiments [42].

### Classification of the test heartbeats using an ensemble of the three classifiers

Following the construction of the minimum distance classifier, the linear discriminant classifier, and the linear SVM, the heartbeats in the testing set DS2 were classified using an ensemble of the three classifiers. The entire classification process is summarized in the following four sections:

1) The preprocessed test heartbeats of the two leads together with the RR intervals formed test feature vectors and were used as the input feature vectors of the minimum distance classifier. The distances from any vector of the test feature vectors to the NORM mean vector and the LBBB mean vector were then calculated and compared, and the test feature vector was assigned to the type with the closer mean vector, namely, NORM or LBBB.

2) The preprocessed test heartbeats from each lead were projected onto the 100 independent components (generated using 620 heartbeats, including NORM, LBBB, and RBBB in the training set) to produce 100 ICA-based features. The ICA-based features of leads A and B were concatenated together and were combined with the RR interval to form the 201-dimensional test feature vectors. The test feature vectors were utilized as the input feature vectors of the weighted linear discriminant classifier. The posterior probabilities of any unknown test feature vector that belonged to NORM and RBBB were calculated by Equations (2) and (3). The unknown test feature vector was then assigned to the type with the maximum posteriori probability, namely, NORM or RBBB.

3) The 201-dimensional test feature vectors previously discussed were also used as the input feature vectors of the linear SVM. The linear SVM produced two possible type labels for a test feature vector, namely, LBBB or RBBB.

4) Finally, with a maximum voting strategy, the votes for the three heartbeat types were counted, and the test feature vectors were then assigned to the type labels with the highest number of votes. When the type labels from the three classifiers were combined, it was possible that three different type labels were produced by these classifiers. In this case, the final type of the heartbeat was assigned to NORM because of the substantially greater quantity of NORM compared with LBBB and RBBB.

### Experimental setup

To assess the performance of the heartbeat classification, classification performance indexes were used. These indexes included the sensitivity (Se), positive predictive value (PP), and accuracy (Acc). The sensitivity refers to the proportion of correctly detected heartbeats of a certain type against all heartbeats of that type. The positive predictive value refers to the proportion of real positive heartbeats of a certain type against all detected heartbeats of that type, and the accuracy refers to the number of correctly identified heartbeats as a percentage of the total heartbeats. When the heartbeat type is NORM, the sensitivity is translated into the specificity (Sp), and the positive predictive value is translated into the negative predictive value (NP). All indexes can be calculated from the confusion matrix.

Several experiments were conducted to construct and evaluate the proposed method. First, the three classifiers were constructed based on each pair of heartbeat types in the training set DS1. Then, the effects of different lead configurations on the classification results for each pair of heartbeat types in the testing set DS2 were assessed, and the final classification results that used an ensemble of the three classifiers were represented. To demonstrate the rationality of using three different classifiers, the performance of these three classifiers was compared for the detection of each pair of heartbeat types, and the classification results of the proposed method were compared with those of the methods that used the same three classifiers. Finally, the rationales for utilizing different classifiers between different pairs of heartbeat types, the feature extraction methods, and the comparison with other methods were discussed.

The ECG data included data from two leads, which were designated lead A and lead B. The features of lead A and lead B were concatenated together when the two leads were applied and were combined with the RR interval to form a feature vector.

For the construction of the minimum distance classifier, the mean vectors of NORM and LBBB were computed based on the training set DS1 without considering parameter selection.

When constructing the weighted linear discriminant classifier, a 10-fold cross-validation on the data that contained NORM and RBBB in the training data set DS1 was used to identify the optimal values of the two weighting coefficients. The training set was divided into 10 parts, with one part left out as a testing set for each cross-validation and the remaining nine parts as a training set. The 10 classification confusion matrices were then summed to calculate the specificity and negative predictive value of NORM and the sensitivity and positive predictive value of RBBB. The benefit of this process was to avoid the error caused by averaging, which reflected the actual classification. Because the number of NORMs was approximately 10 times the number of RBBB heartbeats, the weighting coefficient *w*_1_ of NORM included the values between 0.01 and 0.1, with increments of 0.01, whereas the weighting coefficient *w*_2_ of RBBB included the values 0.4, 0.5, and 0.6. This process produced 30 sets of parameters. For different lead data, the optimal parameters for the linear discriminant classifier were selected from these 30 sets to maximize the RBBB sensitivity. The parameters that maximized the RBBB positive predictive value were selected if the sensitivities were the same.

When the linear SVM was constructed, the previously described 10-fold cross-validation method was used to identify the optimal value of the penalty parameter *C* for the data that contained LBBB and RBBB in the training data set DS1. The 10 classification confusion matrices were then summed to calculate the sensitivity and positive predictive value of LBBB and RBBB. The penalty parameters were set to 0.001, 0.01, 0.1, 1, 10, and 100, and the optimal parameters for different leads were selected from these six sets of parameters based on the mean sensitivity (mean value of the sensitivity of the two heartbeat types). The parameter with the smallest value was selected if the mean sensitivities were the same.

FastICA codes (http://www.cis.hut.fi/projects/ica/fastica) were employed to calculate the independent components. The linear SVM was implemented by liblinear-1.93 (http://www.csie.ntu.edu.tw/~cjlin/liblinear/). The programs were written and run in the MATLAB environment.

## Results

In this study, an improved classification performance was achieved via the construction of an effective classifier between each pair of heartbeat types. The one-against-one classification methods were used to implement a multi-class classification. Therefore, the classification results of each pair of heartbeat types in the testing set under different lead configurations were first represented. Then, the final classification results of the three heartbeat types under the optimum lead configuration were identified by combining the results of each pair of heartbeat types with the maximum voting strategy. To indicate the rationality of using a different classifier for each pair of heartbeat types, the performance comparison of these three classifiers for the detection of each pair of heartbeat types and the performance comparison of the proposed method with the methods of using an ensemble of the same three classifiers were represented, respectively.

It is worth mentioning that we used independent data as a test set to validate the proposed method. The performance indexes of the proposed method were obtained based on the data that were independent of the data used to construct the classifiers and determine the optimum parameters. Furthermore, data as a test set originated from different patients than those providing data used to train the classifiers. Therefore, the results in this study provided a valid assessment of the predictive capability for unknown data.

### Classification of NORM and LBBB under different lead configurations using the minimum distance classifier

**Figure 5:**
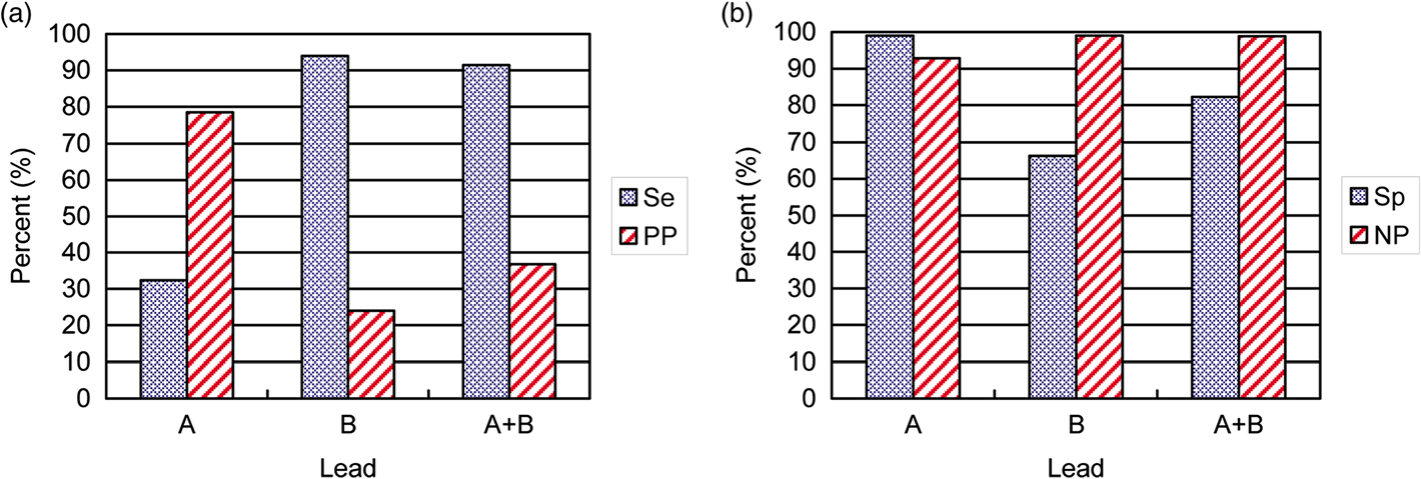
**Comparision of the classification performance of LBBB and NORM under different lead configurations on DS2. (a)** The sensitivity (Se) and positive predictive value (PP) of LBBB under different lead configurations. **(b)** The specificity (Sp) and negative predictive value (NP) of NORM under different lead configurations.

The classification performance for NORM and LBBB in the dataset DS2 under different lead configurations is shown in Figure 5. When configuration lead A was used, LBBB showed a relatively low sensitivity at 32.4%. When configuration lead B was used, the specificity for NORM was 66.3%. When a two-lead configuration was employed, LBBB showed a sensitivity of 91.4% and a positive predictive value of 36.8%, whereas NORM exhibited a specificity of 82.3% and a negative predictive value of 98.8%. The two-lead configuration exhibited an improved classification performance compared with the single-lead configurations. The classification confusion matrix for the two-lead configuration is shown in Table 2. Over 6,000 NORMs were classified as LBBB by mistake, which led to a decreased positive predictive value for LBBB.

**Table 2:** Classification confusion matrix of NORM and LBBB under the two-lead configuration on DS2

**Reference label**	**Algorithm label**		**Total**
	**NORM**	**LBBB**	
**NORM**	29979	6465	36444
**LBBB**	355	3770	4125
**Total**	30334	10235	40569

### Classification of NORM and RBBB under different lead configurations using the weighted linear discriminant classifier

The classification results from the weighted linear discriminant classifier based on Bayesian posterior probability decision making for NORM and RBBB are shown in Table 3; the results of the different lead configurations and the best parameters are also shown. Under the two-lead configuration, RBBB showed a sensitivity of 92.9% and a positive predictive value of 88.4%, whereas NORM yielded a specificity of 98.8% and a negative predictive value of 99.3%, which indicated a satisfactory classification performance. Only a low number of heartbeats were misclassified between NORM and RBBB.

**Table 3:** Classification results of NORM and RBBB under different lead configurations on DS2

**Method**	**Parameters**		**NORM (%)**		**RBBB (%)**		**Acc (%)**
	**W1**	**W2**	**Sp**	**NP**	**Se**	**PP**	
Lead A	0.01	0.4	94.1	95.0	48.1	43.8	90.1
Lead B	0.01	0.6	67.0	93.3	49.8	12.6	65.5
Lead A + B	0.03	0.6	98.8	99.3	92.9	88.4	98.3

### Classification of LBBB and RBBB under different lead configurations using the linear SVM

The LBBB and RBBB classification results from the linear SVM, as well as the results for the different lead configurations and the best parameters are shown in Table 4. Under the two-lead configuration, LBBB and RBBB exhibited excellent classification results, with both the sensitivity and the positive predictive value reaching above 99% for the two heartbeat types. Approximately ten heartbeats were misclassified between LBBB and RBBB.

**Table 4:** Classification results of LBBB and RBBB under different lead configurations on DS2

**Method**	**Parameters**	**LBBB (%)**		**RBBB (%)**		**Acc (%)**
	**C**	**Se**	**PP**	**Se**	**PP**	
Lead A	100	50.3	99.9	99.9	62.9	73.0
Lead B	1	78.4	92.1	92.0	78.2	84.6
Lead A + B	0.1	99.9	99.9	99.9	99.8	99.9

### Final classification performance using ensemble classifiers under the two-lead configuration

According to the classification results between each pair of heartbeat types under different lead configurations, the two-lead configurations were employed to obtain the best classification performance because more classification information was provided by the two-lead data.

Using the three classifiers based on the two-lead data to classify the three types of heartbeats, NORM, LBBB, and RBBB, the final types of heartbeats were obtained through the ensemble of the results from the three classifiers with a majority voting strategy. Table 5 shows the confusion matrix for the heartbeat classification system. The performance indexes were calculated for each heartbeat type based on the information in Table 5, and the results are shown in Table 6. LBBB showed a sensitivity of 91.4% and a positive predictive value of 37.3%, whereas RBBB exhibited a sensitivity of 92.8% and a positive predictive value of 88.8%. The specificity for NORM was 81.5%, and the negative predictive value was 98.0%. The classification performance for the three heartbeat types was all close to the lower classification performance indexes in the pair-wise classification.

**Table 5:** Classification confusion matrix of NORM, LBBB, and RBBB using an ensemble of three classifiers

**Reference label**	**Algorithm label**			**Total**
	**NORM**	**LBBB**	**RBBB**	
**NORM**	29697	6339	408	36444
**LBBB**	355	3770	0	4125
**RBBB**	249	0	3227	3476
**Total**	30301	10109	3635	44045

**Table 6:** The final classification performance of NORM, LBBB, and RBBB on DS2

**NORM (%)**		**LBBB (%)**		**RBBB (%)**	
**Sp**	**NP**	**Se**	**PP**	**Se**	**PP**
81.5	98.0	91.4	37.3	92.8	88.8

In addition, the classification results for the recordings that contained LBBB or RBBB in the testing set DS2 are listed in Table 7. Except for recording 232, a satisfactory classification performance was observed in all cases for LBBB and RBBB.

**Table 7:** Classification performance on each recording of DS2

**Record**	**Number of beats**			**NORM**		**LBBB**		**RBBB**	
	**NORM**	**LBBB**	**RBBB**	**Sp (%)**	**NP (%)**	**Se (%)**	**PP (%)**	**Se (%)**	**PP (%)**
111	0	2123	0	-	0	83.4	100	-	0
212	923	0	1825	99.0	95.4	-	-	97.6	99.5
214	0	2002	0	-	0	99.9	100	-	-
231	314	0	1254	100	99.7	-	-	99.9	100
232	0	0	397	-	0	-	-	48.6	100

### Performance comparison among three different classifiers for classifying each pair of heartbeat types

To demonstrate the construction of the effective classifier between each pair of heartbeat types in this study, the previously trained minimum distance classifier, linear discriminant classifier and linear SVM were employed to classify each pair of heartbeat types in the testing set. The results are shown in Tables 8, 9, and 10, and the classifier with the best performance is expressed in bold.

**Table 8:** Classification performance comparison of NORM and LBBB using three classifiers

**Method**	**NORM (%)**		**LBBB (%)**	
	**Sp**	**NP**	**Se**	**PP**
**Minimum distance classifier**	82.3	98.8	91.4	36.8
Linear discriminant classifier	70.6	90.6	35.2	11.9
Weighted linear SVM	77.3	98.7	91.2	31.2

The classifier with the best performance is expressed in bold. The feature vector was composed of the preprocessed heartbeat signals from both leads and a RR interval.

**Table 9:** Classification performance comparison of NORM and RBBB using three classifiers

**Method**	**NORM (%)**		**RBBB (%)**	
	**Sp**	**NP**	**Se**	**PP**
Minimum distance classifier	73.6	88.9	4.1	1.5
**Linear discriminant classifier**	98.8	99.3	92.9	88.4
Weighted linear SVM	94.8	99.0	89.9	62.0

The classifier with the best performance is expressed in bold. The feature vector was composed of the ICA features from both leads and a RR interval.

**Table 10:** Classification performance comparison of LBBB and RBBB using three classifiers

**Method**	**LBBB (%)**		**RBBB (%)**	
	**Se**	**PP**	**Se**	**PP**
Minimum distance classifier	99.8	99.2	99.0	99.7
Linear discriminant classifier	99.7	99.6	99.6	99.6
**Linear SVM**	99.9	99.9	99.9	99.8

The classifier with the best performance is expressed in bold. The feature vector was composed of the ICA features from both leads and a RR interval.

As shown in Table 8, the minimum distance classifier exhibited the best classification performance between NORM and LBBB, followed by the weighted linear SVM (i.e., the weighted linear SVM was required because the number of heartbeats that were classified as LBBB was far less compared with the number of NORMs). The weighted linear SVM is described as follows. If the numbers of two heartbeat types to be classified are imbalanced, then to avoid results skewed towards the majority class, different penalties can be introduced for each type in the objective function. Thus, the penalty parameter and weighting factors were experimentally determined to achieve the best classification performance. The linear discriminant classifier showed the worst results between NORM and LBBB.

As shown in Table 9, the linear discriminant classifier showed the best performance in the classification of NORM and RBBB, followed by the weighted linear SVM. LBBB and RBBB showed good separability. The linear SVM exhibited the best performance in the classification of LBBB and RBBB, followed by the linear discriminant classifier (see Table 10).

The results indicated that the classifier utilized between each pair of heartbeat types in this study had the best classification performance among the three classifiers.

### Comparison with the methods of using an ensemble of three identical classifiers

To demonstrate that ensembles of three different classifiers can significantly improve the classification performance in heartbeat classification, the results of combining the three different classifiers were compared with the results of combining three identical classifiers as shown in Table 11. The proposed method demonstrated the best classification performance. These results indicated that an improved classification performance can be achieved by constructing classifiers with the best performance between each pair of heartbeat types. The findings also indicated the complexity of heartbeat classification, and a single classifier is not sufficient to obtain good heartbeat classification results. To achieve better performance, different classifiers are required when the distribution and extent of overlap between every two types of heartbeats are different.

**Table 11:** Classification performance comparison of the proposed method with the methods using the same three classifiers

**Method**	**NORM (%)**		**LBBB (%)**		**RBBB (%)**	
	**Sp**	**NP**	**Se**	**PP**	**Se**	**PP**
Minimum distance classifier	55.9	84.7	91.4	36.9	4.1	1.5
Linear discriminant classifier	77.0	90.6	35.2	15.3	92.8	89.6
(Weighted) linear SVM	72.1	97.4	91.1	31.3	89.9	62.1
Proposed method	81.5	98.0	91.4	37.3	92.8	88.8

## Discussion

We first explained why the different classifiers were used between different pairs of heartbeat types. Then, we discussed the selection of the input features for the three classifiers and the comparison with other methods.

### The rationale for utilizing different classifiers between different pairs of heartbeat types

We constructed different classifiers between different pairs of heartbeat types and obtained satisfactory classification performance. This finding can be explained by the distribution and extent of overlap between every two types of heartbeats. Because the NORMs originated from 22 patients in each dataset and the number of NORMs was very high, i.e., up to tens of thousands, the NORMs showed large variations and thus generated overlapping beats between NORM and other heartbeat types. Through our careful observation in the experiments, the morphologies and RR intervals of a part of the LBBB and RBBB beats were similar to those of the NORMs. Therefore, there were overlapping beats at the border between LBBB and NORM and between RBBB and NORM. Furthermore, the distribution and extent of overlap between every two types of heartbeats were different.

LBBB and NORM are particularly difficult to distinguish because NORM and LBBB overlap significantly, and the distribution of LBBB is not close to a Gaussian distribution. The linear discriminant classifier that assumed the data conformed to a Gaussian distribution was used to classify the heartbeats and achieved the worst results. The minimum distance classifier was used for the classification of NORM and LBBB according to the distances of any heartbeat in the testing set to the centroids of two heartbeat types. This approach can reduce the misclassification of these border heartbeats between the two types more effectively compared with the weighted linear SVM. The weighted linear SVM handles the overlapping beats through the penalty parameter and weighting factors. As a result of the significant overlap between NORM and LBBB, the weighted linear SVM has limitations with respect to the nonlinear separable case.

The distributions of RBBB and NORM were close to a Gaussian distribution, which satisfied the assumption required by the linear discriminant classifier. Therefore, the linear discriminant classifier could effectively eliminate the impact from the overlapping beats at the boundary of the two types of heartbeats and thus obtained good results. As a result of the good separability of LBBB and RBBB, the three classifiers all exhibited good classification performance, but the linear SVM had an advantage for handling the error-classifying samples through the penalty parameter and showed the best performance.

With respect to the classifier ensemble, the combination of multiple classifiers can facilitate the integration of knowledge obtained from different classifiers and thus improve the classification performance [30,31]. To obtain satisfactory ensemble classification results, the component classifiers are required to possess a good classification performance and simultaneously be different from each other [32].

In summary, we should choose different classifiers between different pairs of heartbeat types to attain the best performance because the distribution and extent of overlap between every two types of heartbeats are different, and the classifier ensemble also requires different component classifiers to obtain a good ensemble classification performance.

### Consideration of feature extraction

The classification performance is not only related to the classifier used but is also affected by feature extraction. Feature extraction is aimed at achieving better classification performance with a reduced number of feature dimensions, which increases the classification speed. Parameter estimation is the most common method for heartbeat feature extraction, and it typically produces a lower number of feature vector dimensions. However, these parameters are affected by the human body and instrument noise, which results in variations in different patients. The extraction of parameter features relies on accurate ECG measurement [29], and thus, this feature extraction method was not adopted in this study. The direct method uses the amplitude of ECG signals, namely, the morphological features as the feature vector to achieve a low computational cost but with a higher number of dimensions.

Compared with morphological features, transform methods can extract information that cannot be readily obtained from the original signals. Among the transform methods, linear discriminant analysis (LDA), PCA, and ICA can prevent the divergence of feature vectors in the feature space and simultaneously reduce dimensions. Due to the overlap between NORM and RBBB, the use of LDA does not provide adequate separation [43]. Although there is no clear overlap between LBBB and RBBB, the use of ICA has obtained good classification results, and thus, we did not use LDA to extract the features of the heartbeats. Compared with PCA, ICA captures both second and higher order statistics and thus contains more feature information. For these reasons, the features used in the classification between NORM and RBBB and between LBBB and RBBB in this study were all ICA features, and 400-dimension morphological features were transformed to 200-dimension features. Satisfactory classification results were achieved using these features.

However, ICA features were not employed in the classification between NORM and LBBB, and the 400-dimension morphological features were used instead. As shown in Table 12, feature extraction using ICA did not achieve good classification performance, which was likely caused by the severe overlap between NORM and LBBB. The use of ICA made the separation of overlapping samples more difficult. Consequently, morphological features were employed to distinguish between NORM and LBBB. Despite a higher number of dimensions, the computational efficiency was not affected due to the simple computation associated with the minimum distance classifier. LBBB exhibited a low positive predictive value, and thus, further studies are needed to identify features that can accurately identify LBBB.

**Table 12:** Classification performance comparison of the morphological and ICA features for NORM and LBBB

**Method**	**NORM (%)**		**LBBB (%)**	
	**Sp**	**NP**	**Se**	**PP**
Preprocessed heartbeat	82.3	98.8	91.4	36.8
ICA features	97.4	96.0	63.9	73.9

### Comparison with other classification methods

The aim of this paper was to explore an inter-patient classification method for automatically detecting LBBB and RBBB from NORM. To our knowledge, few studies have been reported on the detection of LBBB and RBBB based on inter-patient classification. Nevertheless, we still compared the results of the proposed method with other intra-patient classification methods. Whether the methods belonged to the inter-patient classification is shown in Table 13. These methods included the time-frequency beat descriptors and the kth nearest neighbor classification [7]; parameter features and the kth nearest neighbor classification [8]; a feed forward back propagation neural network using higher order statistics of wavelet transform subband components [9]; SVM classification using power spectral features [10]; the nearest neighbor classification using local fractal dimension [11]; SVM classification using the wavelet with best discrimination capability [12]; and probabilistic neural network with a feature reduction method [13]. All intra-patient methods obtained higher detection performance for LBBB and RBBB compared with the proposed method, except for the methods proposed in [8] and [12]. These two methods used a small-size training set; thus, their performance was affected. Because intra-patient classification methods were trained on the non-independent training datasets, obtaining a good predictive performance for LBBB and RBBB was difficult. They can be used for adaptive-patient heartbeat classification to improve classification performance but require expert annotation.

**Table 13:** Performance comparison of the proposed method with other methods

**Method**	**Inter-patient**	**NORM (%)**		**LBBB (%)**		**RBBB (%)**	
		**Sp**	**NP**	**Se**	**PP**	**Se**	**PP**
Christov [7]	No	96.9	98.4	95.7	99.2	94.4	99.3
Jekova [8]	No	94.8	98.1	58.1	74.4	88.5	78.9
Yu [9]	No	98.8	-	98.8	-	99.2	-
Khazaee [10]	No	94.3	99.4	98.9	98.5	98.9	98.8
Mishra [11]	No	98.9	99.6	97.4	97.5	97.9	98.5
Daamouche [12]	No	86.3	-	88.8	-	89.4	-
Wang [13]	No	99.6	99.8	98.8	99.5	99.3	100
Yeh [26]	Unknown	99.0	97.3	91.1	96.5	95.1	94.2
Yeh [27]	Unknown	98.3	97.4	90.4	91.0	87.0	87.1
Yeh [28]	Unknown	95.6	97.9	91.3	92.3	90.5	90.7
Jekova [8]	Yes	87.2	92.3	18.8	25.2	43.2	52.7
Mishra [11]	Yes	93.2	-	87.4	-	82.4	-
Dokur [29]	Yes	100	96.7	94.6	91.0	98.6	94.2
Proposed method	Yes	81.5	98.0	91.4	37.3	92.8	88.8

Yeh et al. [26-28] studied the classification of LBBB, RBBB, and NORM and proposed several classification methods based on a group of recordings in the MIT-BIH Arrhythmia Database, which showed high classification performance as shown in Table 13. These methods chose four qualitative features using a range-overlap method and adopted a fuzzy logic method, LDA, and cluster analysis for classification, respectively. However, it is not clear whether the dataset for building the classification model was independent from the testing set. Furthermore, the recordings used in their study were different from the recordings used in current study. Fourteen recordings were used in their study. In addition, the numbers of RBBB and NORM in two recordings, 212 and 231, were different from their numbers in this study. We used the class labels in the annotation files of the MIT-BIH Arrhythmia Database. Therefore, due to the different datasets used, the proposed method showed a different classification performance compared with the methods proposed by Yeh et al. [26-28]. Their methods did not explicitly designate the independent training set and thus were not counted as inter-patient classification.

Inter-patient classification methods were developed using independent training datasets and thus can be used to predict LBBB and RBBB from the unknown patients’ ECG recordings. Furthermore, they do not require expert annotation and can perform detection automatically. Jekova et al. [8] and Mishra et al. [11] also performed inter-patient heartbeat classification, but the results were inferior to those based on intra-patient classification shown in Table 13. Jekova et al. [8] adopted 26 morphological parameters for feature extraction; the use of low-dimension features can lead to a decrease in the operating time of the kth nearest neighbor classifier. However, the parameter features are affected by the human body and instrument noise. Jekova et al. selected 1 heartbeat of each available type from each record, which resulted in 91 heartbeats in the training set. When each recording was classified, only the training heartbeats from the remaining 47 recordings were used. The method cannot make full use of the information of more heartbeats and produced worst results, i.e., a sensitivity of 18.8% for LBBB and a sensitivity of 43.2% for RBBB. Mishra et al. [11] utilized power spectral density based fractal dimensions for feature extraction, which can prevent the divergence of feature vectors in the feature space. They used 11 recordings obtained from 25 recordings as the testing set, and the remaining 14 recordings were used as the training set. The average sensitivities of LBBB and RBBB were 87.4% and 82.4%, respectively, but the positive predictive values were not provided. The performance of this method was lower compared with our method because using only one classifier cannot yield better results given a complicated heartbeat distribution. Dokur et al. [29] proposed an intersecting sphere (Ins) network and discrete wavelet transform for inter-patient classification. The InS network has a high generalization ability, and the dimension of the feature vector is only 8, contributing to an improvement in the operating speed. This method yielded a higher performance compared with the proposed method, a sensitivity of 94.6% for LBBB and a sensitivity of 98.6% for RBBB. However, because each heartbeat type had only 150 heartbeats separately in the training and testing sets, it reduced the difficulty of classification and led to optimistic results.

The proposed method was constructed and evaluated based on inter-patient classification schema. We achieved satisfactory classification results by constructing an effective classifier between each pair of heartbeat types according to the different extents of overlap and different distributions between every two types of heartbeats. The proposed method is better compared with the method proposed by Mishra et al. [11], which benefits from adopting the ensemble classifiers. Furthermore, we used 22 recordings as the training set and 22 recordings as the testing set, which made complete use of the heartbeats to train the classification model and provided more objective results. The method can be ported to a real-life heartbeat classification system with some fine-tuning. However, prior to applying the proposed method, the algorithm for classifying class N beats following AAMI is required because our method was developed based on class N beats, which primarily included NORM, LBBB, and RBBB. Again, the proposed method employed the ensemble of three different classifiers to improve the detection performance, but it also increased the complexity of the entire algorithm. However, the computational speed of computers is currently fast, thus, this is not a problem. The proposed method is promising for clinical applications.

Due to the different datasets used, it is difficult to perform a fair and objective comparison based purely on the classification results. Nevertheless, the results from this study can be deemed an objective reflection of the predictive capability of unknown data because the testing set was not used in the prior construction of the training model in this study.

## Conclusions

Inter-patient classification is critical in improving the predictive capability of a heartbeat classification system. To accurately distinguish LBBB and RBBB from NORM, this study proposed a heartbeat classification method that combined three different classifiers. This method was based on inter-patient data division and achieved high performance classification by constructing an effective classifier between each pair of the heartbeat types examined. Using different classifiers is conducive to constructing better classification models according to the different extents of overlap and different distributions of each pair of sample types. In this study, we constructed a minimum distance classifier between NORM and LBBB, a weighted linear discriminant classifier between NORM and RBBB based on Bayesian posterior probability, and a linear SVM between LBBB and RBBB. Satisfactory classification results were obtained by combining the three classifiers using a majority voting strategy, with a sensitivity of 91.4% and a positive predictive value of 37.3% for LBBB and a sensitivity of 92.8% and a positive predictive value of 88.8% for RBBB. Because the classification model selected was entirely based on the training set and the final classification performance was assessed using the best configuration on the independent testing set, the results from this study can be deemed an objective reflection of the predictive capability for unknown data. The proposed heartbeat classification method has the potential for clinical use.

## Abbreviations

LBBB: Left bundle branch block; RBBB: Right bundle branch block; ECG: Electrocardiogram; NORM: Normal beat; AAMI: Association for the Advancement of Medical Instrumentation; SVM: Support vector machine; ICA: Independent component analysis; PCA: Principal component analysis; RBF: Radial basis function; Se: Sensitivity; PP: Positive predictive value; Acc: Accuracy; Sp: Specificity; NP: Negative predictive value; LDA: Linear discriminant analysis.

## Competing interests

The authors declare that they have no competing interests.

## Authors’ contributions

HH and GH conceived of the study and designed the experiments. HH performed the experiments. All authors participated in the analysis of data. HH and GH wrote the manuscript. All authors read and approved the final manuscript.

## Authors’ information

HH: She is currently a lecturer at Department of Biomedical Engineering, Beijing Jiaotong University, Beijing, China. She got her Master degree and PhD degree from the Department of Biomedical Engineering, School of Medicine, Tsinghua University, Beijing, China. She has about over 10 years of research experience in Medical Pattern Recognition, including feature extraction and classifier design. She has performed study about iris recognition and ECG heartbeat classification. Now she conducts research work about the psychiatric disorder multivariate pattern classification based on fMRI, such as ADHD and depression.

JL: He is a Professor in the Department of Biomedical Engineering, Beijing Jiaotong University, Beijing, China. His research interests are Biomedical Signal/Image Processing and Medical Imaging.

QZ: She is an Associate Professor in the Department of Biomedical Engineering, Beijing Jiaotong University, Beijing, China. Her research interests are Biomedical Signal Processing.

RW: She is an Associate Professor in the Department of Biomedical Engineering, Beijing Jiaotong University, Beijing, China. Her research interests are Biomedical Signa/Image Processing and Medical Pattern Recognition.

GH: He is a Professor in the Department of Biomedical Engineering, School of Medicine, Tsinghua University, Beijing, China. His research interests are Biomedical Signal/Image Processing, Medical Pattern Recognition and Digital Medical Instrumentation. His research work is focused on the detection and processing of ECG, EEG and evoked potential signals, X-ray image cardio-cerebral angiography, CT and MRI images, iris images, multifocal electro-oculography feature extraction and clinical application, the application of DSP in clinical instruments etc.
